# Alternative RNA Splicing in Cancer: Molecular Mechanisms, Functional Consequences, Biomarkers and Therapeutic Opportunities

**DOI:** 10.3390/genes17090984

**Published:** 2026-08-22

**Authors:** Quanyou Wu, Kai Gui

**Affiliations:** 1 Laboratory of Molecular Targeted Therapy in Oncology, Division of Abdominal Cancer, Department of Medical Oncology, Cancer Center, West China Hospital, Sichuan University, Chengdu 610041, China; 2 Key Laboratory of Clinical Laboratory Diagnostics, Ministry of Education, School of Laboratory Medicine, Chongqing Medical University, Chongqing 400016, China; gktoudingkan@126.com

**Keywords:** alternative splicing, spliceosome, RNA-binding proteins, cancer, antisense oligonucleotides

## Abstract

Alternative pre-mRNA splicing is a central layer of gene regulation that enables a limited number of genes to generate a far larger and more context-dependent transcriptome and proteome. In cancer, splicing is disrupted by mutations in cis-regulatory sequences, recurrent lesions in spliceosome components, altered abundance or activity of RNA-binding proteins, and changes in transcription, chromatin, RNA modification, metabolism and stress signalling. These alterations are not merely by-products of malignant transformation. They can create oncogenic protein isoforms, eliminate tumour-suppressive products, remodel cellular identity, promote metastasis and drug resistance, and generate tumour-restricted peptides that are visible to the immune system. Large pan-cancer datasets, long-read sequencing, single-cell isoform profiling, proteogenomics and functional perturbation screens are now resolving this complexity at unprecedented scale. In parallel, multiple therapeutic strategies are advancing, including modulators of the SF3B complex, molecular glues that degrade RBM39, inhibitors of protein arginine methyltransferases and splicing kinases, splice-switching oligonucleotides, programmable RNA-targeting systems, and vaccines or T-cell receptors directed against splicing-derived neoantigens. This review integrates the molecular logic of splice-site selection with the cancer-specific mechanisms that perturb it, summarizes representative isoform switches across the hallmarks of cancer, evaluates emerging technologies and clinical biomarkers, and discusses the opportunities and constraints of translating splicing biology into precision oncology. Particular emphasis is placed on tumour specificity, intratumoural heterogeneity, proteomic validation, therapeutic windows and rational combination strategies.

## 1. Introduction

Alternative pre-mRNA splicing (AS) is the regulated selection of exons, introns and splice sites from a nascent RNA precursor. It allows one gene to produce transcripts that differ in coding sequence, untranslated regions, localization signals, RNA stability and translational efficiency. Most human multi-exon genes are alternatively spliced, and many genes produce dozens of detectable transcripts, although only a subset is abundant, conserved or translated in any particular cell type [1,2,3,4,5,6,7,8]. The biological meaning of splicing therefore lies not simply in transcript number, but in the controlled redistribution of isoforms across development, differentiation, stress and disease.

Cancer cells exploit this regulatory layer in several ways. They may acquire mutations that create or disrupt splice signals, select recurrent mutations in core spliceosome proteins, overexpress oncogenic splicing factors, or rewire transcriptional and epigenetic programs that change splice-site recognition. The resulting isoforms can alter entire protein domains, interaction motifs, enzymatic activity, membrane topology or subcellular localization. Other events introduce premature termination codons and trigger nonsense-mediated decay (NMD), thereby reducing gene dosage without changing the DNA copy number. These mechanisms intersect with essentially every hallmark of cancer, including sustained proliferation, resistance to cell death, metabolic adaptation, invasion, immune evasion, lineage plasticity and therapeutic resistance [9,10,11,12,13,14,15,16].

Several observations have moved splicing from a descriptive transcriptomic phenomenon to a mechanistic and translational field. First, recurrent mutations in *SF3B1*, *SRSF2*, *U2AF1*, *ZRSR2* and U1 small nuclear RNA produce stereotyped splice signatures that can be causally linked to malignant phenotypes [17,18,19,20,21,22,23,24,25,26,27]. Second, pan-cancer analyses have identified thousands of tumour-enriched or tumour-specific splice junctions, many of which are not explained by coding mutations [17,28,29,30,31]. Third, long-read and single-cell technologies now resolve full-length transcript architecture and cell-state-specific isoform usage that short reads cannot reliably reconstruct [32,33,34,35,36,37,38,39]. Finally, aberrant junctions can generate public, clonally distributed neoantigens that are naturally presented by major histocompatibility complex (MHC) molecules and recognized by T cells [37,40,41,42,43].

The central challenge is selectivity. Constitutive splicing is essential in normal tissues, while many cancer-associated events are quantitative rather than binary and vary among clones, anatomical sites and treatment states. A clinically useful target must therefore combine biological dependency with adequate tumour restriction, protein-level evidence, delivery feasibility and a therapeutic window. This review describes the multilayer regulation of pre-mRNA splicing, the origins and functions of its dysregulation in cancer, the technologies required to characterize isoforms rigorously, and the emerging therapeutic strategies that exploit either the splicing machinery or individual cancer-associated transcripts.

## 2. Molecular Basis and Regulation of Pre-mRNA Splicing

### 2.1. The Spliceosome and the Catalytic Cycle

The major spliceosome is a dynamic ribonucleoprotein machine composed of U1, U2, U4/U6 and U5 small nuclear ribonucleoproteins (snRNPs) together with more than one hundred associated proteins. It recognizes a 5′ splice site, a branch point adenosine, a polypyrimidine tract and a 3′ splice site, then catalyzes two transesterification reactions that remove the intron as a lariat and ligate the flanking exons [1,2,3,4]. Assembly proceeds through ordered but highly dynamic complexes. U1 initially engages the 5′ splice site, SF1 and U2AF proteins help define the branch point and 3′ region, U2 replaces SF1 at the branch point, and the U4/U6.U5 tri-snRNP is recruited before extensive rearrangements create the catalytically active centre. ATP-dependent RNA helicases proofread these transitions and promote disassembly after exon ligation [2,3].

The minor spliceosome processes a small but biologically important set of U12-type introns using U11, U12, U4atac and U6atac together with U5. Because many U12-type introns occur in genes controlling DNA replication, cell-cycle progression and RNA processing, disruption of the minor spliceosome can have disproportionate biological consequences. *ZRSR2* mutations, which preferentially impair U12-type intron removal, illustrate how a numerically small intron class can become a recurrent vulnerability in myeloid neoplasms [19,25].

**Figure 1:**
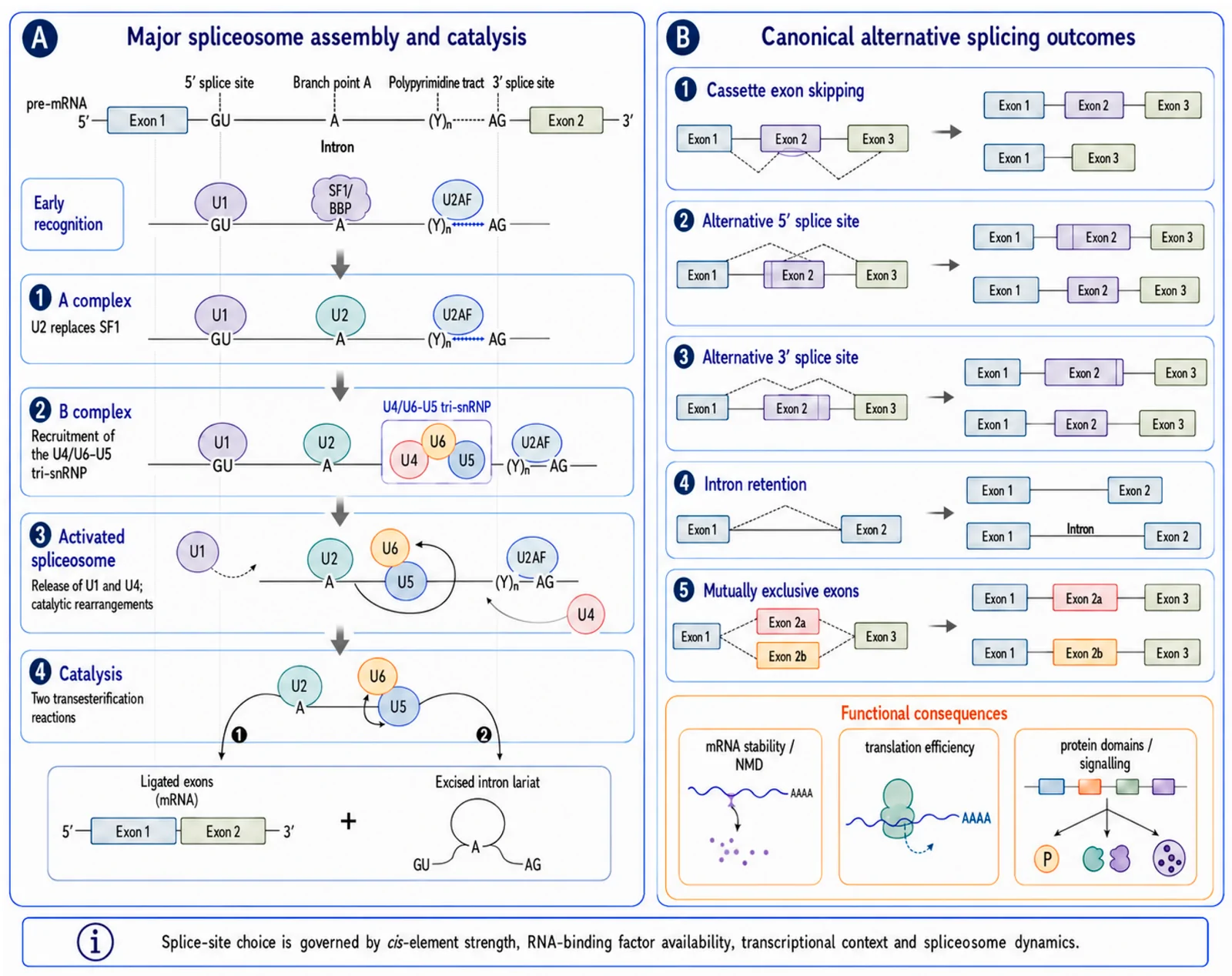
Overview of major spliceosome assembly and canonical alternative pre-mRNA splicing. (**A**) The major spliceosome recognizes the 5′ splice site, branch point and 3′ splice site through sequential assembly of U1, U2 and the U4/U6·U5 tri-snRNP, followed by catalytic rearrangement, intron-lariat excision and exon ligation. (**B**) Canonical alternative-splicing modes include cassette exon skipping, alternative 5′ and 3′ splice-site selection, intron retention and mutually exclusive exons. These decisions can alter mRNA stability and nonsense-mediated decay, translational efficiency and protein-domain composition.

Splice-site recognition is a regulated and context-dependent process rather than being determined solely by splice-site sequence. Canonical consensus sequences are short and degenerate, and their information content is insufficient to specify exon boundaries in isolation. Productive recognition emerges from combinatorial interactions among splice-site strength, RNA motifs and structure, the abundance and activity of trans-acting proteins, transcriptional kinetics and local chromatin. This explains why weak splice sites are particularly sensitive to changes in regulatory-factor availability and why a mutation far from a canonical dinucleotide can alter exon inclusion. The major spliceosome assembly pathway and canonical alternative-splicing outcomes are summarized in Figure 1.

### 2.2. Modes and Functional Outputs of Alternative Splicing

The classical AS modes are cassette exon inclusion or skipping, mutually exclusive exons, alternative 5′ splice-site selection, alternative 3′ splice-site selection and intron retention. Alternative first and last exons couple splicing to promoter choice and polyadenylation, while poison exon inclusion can introduce a premature stop codon and regulate the abundance of splicing factors through NMD. More complex events include recursive splicing, microexons, detained introns, alternative terminal coding sequences and coordinated changes across multiple exons [5,6,7,8].

Isoform switching can change protein function at several levels. Inclusion or exclusion of a catalytic domain may create active and inactive enzymes; membrane-spanning exons can determine whether a receptor is membrane bound or soluble; short motifs can alter protein–protein interactions, degradation, phosphorylation or localization; and changes in untranslated regions can redirect mRNA stability, translation or subcellular transport. Some alternative transcripts are not productively translated and instead act as a mechanism of gene-dosage control. Consequently, transcript-level changes should not automatically be interpreted as protein isoforms without evidence from ribosome profiling, proteomics or targeted validation [8].

A single change in splice-site choice can therefore exert broader effects than an isolated change at the transcriptional or translational level because it may simultaneously alter RNA dosage, translation and protein architecture. For example, *MDM4* poison exon inclusion introduces a premature termination codon and channels the transcript to nonsense-mediated decay, thereby lowering *MDM4* abundance [44]; *BCL2L1* and *MCL1* splice switches generate proteins with opposing pro- and anti-apoptotic activities [45,46]; and *MET* exon 14 skipping removes the CBL-binding region, stabilizing MET protein and creating a clinically actionable oncogenic state [47]. These examples illustrate how a focal splicing defect can propagate through RNA stability, protein-domain composition and signalling without requiring a change in promoter activity or a new coding mutation.

### 2.3. Cis Elements, RNA-Binding Proteins and Regulatory Networks

Splicing enhancers and silencers are located within exons and introns. Serine/arginine-rich (SR) proteins often bind enhancer elements and promote exon definition by recruiting spliceosomal components, whereas heterogeneous nuclear ribonucleoproteins (hnRNPs) frequently antagonize splice-site recognition or remodel RNA structure. These simplified rules are context dependent: The same factor can promote inclusion at one site and skipping at another according to binding position, neighbouring proteins, RNA secondary structure and transcriptional timing [5,6,48,49]. Tissue-restricted regulators such as RBFOX, NOVA, PTBP, ESRP, MBNL and QKI establish cell-identity programs, while broadly expressed factors such as SRSF1, SRSF2, hnRNPA1 and hnRNPH can be repurposed by oncogenic signalling.

Splicing factors are embedded in homeostatic networks. Many SR proteins and hnRNPs regulate their own poison exons, NMD sensitivity or nuclear export, creating feedback loops that buffer protein abundance. Cancer may break these loops through amplification, altered phosphorylation, mutations in autoregulatory exons or changes in NMD. Because one factor can bind thousands of transcripts, modest changes in its abundance can produce coordinated but selective effects, particularly among weak exons that sit close to a recognition threshold. This network behaviour helps explain why cancer-associated splicing programs can be reproducible even when no single splice event is universally dominant.

### 2.4. Co-Transcriptional Splicing, Chromatin and RNA Modification

Most splicing occurs co-transcriptionally. The carboxy-terminal domain of RNA polymerase II recruits RNA-processing factors, and elongation rate influences the time available for competing splice sites to be recognized. Slow elongation may favour inclusion of a weak exon by extending its recognition window, whereas rapid elongation can promote skipping, although the direction depends on local architecture and factor recruitment [50]. Promoter identity, enhancer activity, transcriptional pausing and three-dimensional genome organization can therefore influence isoform output independently of total gene expression.

Chromatin provides another layer of coupling. Histone modifications can recruit adaptor proteins that interact with splicing factors, and nucleosome occupancy is enriched around exons, potentially affecting polymerase kinetics and exon definition. SF3B1 itself associates with chromatin, and changes in DNA methylation or histone marks can redistribute splice-regulatory complexes [51,52,53]. These mechanisms are important in cancer because mutations in chromatin modifiers, enhancer rewiring and widespread epigenetic plasticity can generate splicing changes without direct lesions in splicing genes.

RNA modifications also influence splicing. Nuclear m6A readers such as YTHDC1 and hnRNPA2B1 can recruit or antagonize splice regulators, while m6A-dependent structural switches expose or occlude protein-binding motifs [54,55,56,57]. The effects are transcript- and context-specific, but they connect metabolic state and methyl-donor availability to RNA processing. This coupling becomes relevant when tumours alter one-carbon metabolism, methyltransferase activity or RNA-modification enzymes.

## 3. Origins of Splicing Dysregulation in Cancer

### 3.1. Cis-Acting Mutations and Structural Alterations

**Figure 2:**
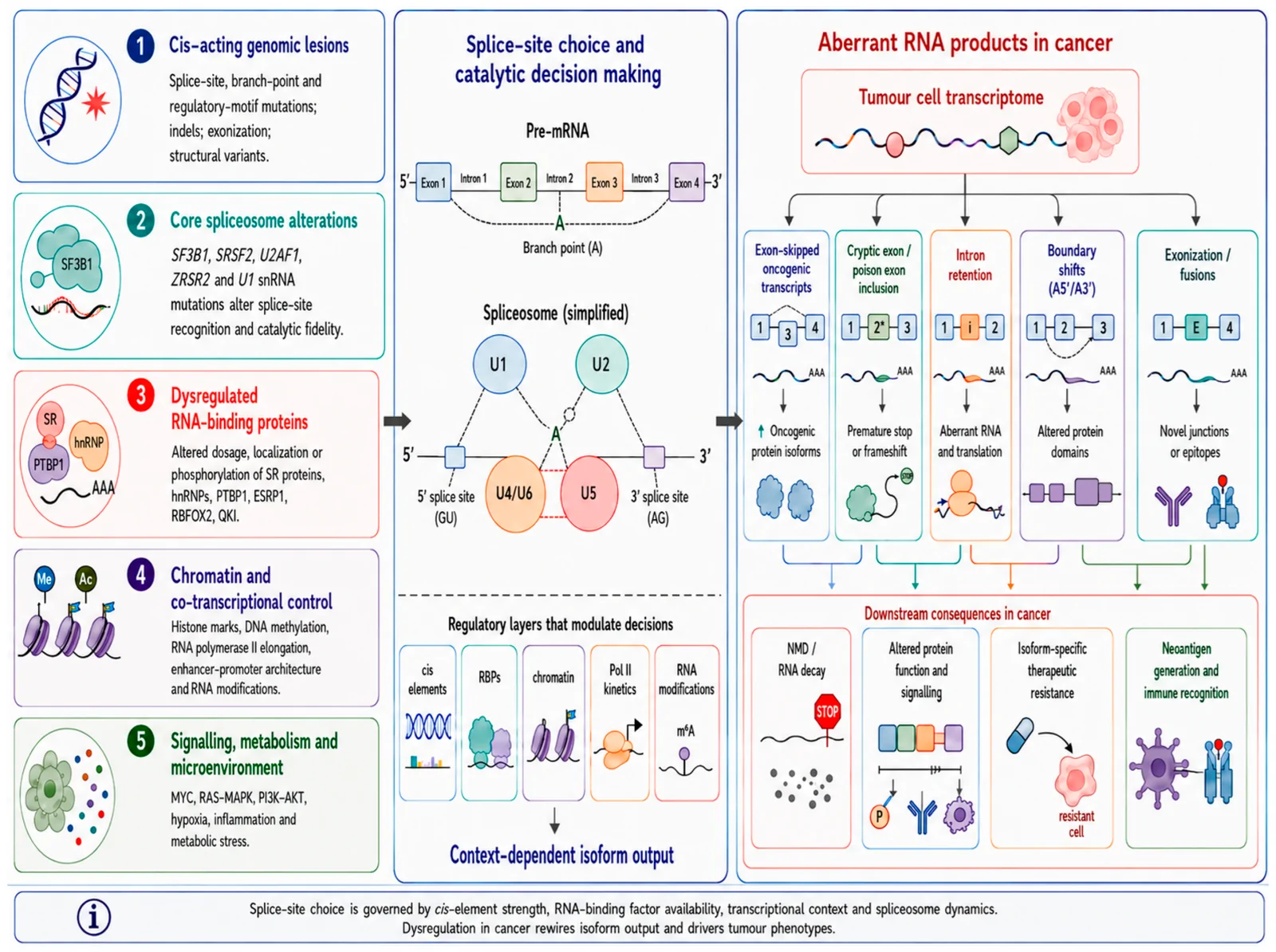
Multilayer architecture and origins of cancer-associated RNA splicing dysregulation. Cis-acting genomic lesions, core spliceosome mutations, dysregulated RNA-binding proteins, chromatin and co-transcriptional control, and signalling or microenvironmental cues converge on splice-site choice and catalytic fidelity. Aberrant outputs include exon skipping, alternative 5′ or 3′ splice-site usage, intron retention and cryptic or poison exon inclusion, leading to RNA decay, altered protein domains and tumour-restricted neoantigens. The red star in the upper-left panel denotes cis-acting genomic lesions.

Cancer-associated splice defects can be classified into cis-acting lesions, trans-acting defects and state-dependent remodelling (Figure 2). Cis lesions include substitutions or indels at the invariant splice-site dinucleotides, branch points, polypyrimidine tracts and enhancer or silencer motifs. They may cause exon skipping, intron retention, cryptic-site activation or pseudoexon inclusion. Synonymous and missense mutations can also alter splicing by changing exonic regulatory motifs, so the functional consequence of a coding variant cannot always be inferred from its amino-acid substitution alone [31,58,59].

Structural variants and copy-number changes can create fusion transcripts, juxtapose cryptic splice sites or alter the dosage of splice regulators. Splice-site-creating mutations are widespread across cancer genomes and can activate latent exons or produce novel C termini [31]. Clinically, *MET* exon 14 skipping is a paradigmatic example: diverse mutations around the exon and adjacent intronic sequences disrupt recognition, remove the CBL-binding region and stabilize the MET receptor, thereby creating sensitivity to MET inhibitors [47]. AR-V7 in prostate cancer, EGFRvIII in glioblastoma and multiple FGFR variants further illustrate how alternative transcript architecture can define therapy response even when the canonical gene remains expressed [60,61,62].

A key analytical issue is whether the observed event is tumour-specific. Normal tissues use many low-abundance isoforms, and incomplete reference annotations can make physiological transcripts appear novel. Comparisons against large normal-tissue panels, matched adjacent tissue, and lineage-matched controls are therefore essential. Additional evidence should include junction-spanning reads, full-length transcript structure, allelic linkage to the causal mutation, and preferably protein or functional validation.

### 3.2. Recurrent Mutations in Core Spliceosome Components

Recurrent mutations in *SF3B1*, *SRSF2*, *U2AF1* and *ZRSR2* are especially prevalent in myelodysplastic syndromes, chronic myelomonocytic leukemia, acute myeloid leukemia and chronic lymphocytic leukemia, with selected occurrences in uveal melanoma, breast cancer, lung cancer and other solid tumours [19,20,21,22,23,24,25,26,27]. These mutations are usually heterozygous and mutually exclusive, suggesting that partial alteration rather than complete loss of splicing is selected. The mutant proteins retain essential function while imposing a new sequence preference or reducing fidelity, producing a characteristic but incomplete splice program.

*SF3B1* hotspot mutations alter branch-point recognition and favour upstream cryptic 3′ splice sites. Many resulting transcripts contain premature stop codons and undergo NMD, whereas others encode altered proteins. The affected targets vary by lineage, which helps explain why the same mutation is associated with distinct phenotypes in myeloid disease, uveal melanoma and solid tumours [21,22,23,24,25]. *SRSF2* mutations change RNA-binding preference, commonly increasing recognition of CCNG over GGNG motifs, and thereby remodel exon inclusion across genes involved in epigenetic control, differentiation and signalling. *U2AF1* mutations alter sequence preferences near the 3′ splice site, while *ZRSR2* loss preferentially impairs U12-type intron removal [19,25,26].

These mutations generate both liabilities and adaptive advantages. Mutant cells operate closer to the limit of spliceosomal capacity and can be hypersensitive to further pharmacological perturbation [27,63,64]. At the same time, mutant-specific mis-splicing can alter inflammatory signalling, DNA damage responses, mitochondrial function and cell differentiation. Their phenotypic output depends on genetic co-mutations, lineage and the selective pressure imposed by therapy. A splice signature is therefore best understood as a distributed network phenotype rather than a single driver transcript.

### 3.3. Dysregulated Rna-Binding Proteins and Oncogenic Transcription

Many tumours lack recurrent spliceosome mutations but alter the abundance or activity of RNA-binding proteins (RBPs). *SRSF1* is a prototypical splicing-factor oncogene: increased expression can transform mammary epithelial cells, promote proliferation and survival, and cooperate with KRAS-driven tumorigenesis [65,66,67,68,69]. SRSF1-dependent programs regulate BCL2L1, MCL1, BIN1, RON, and multiple metabolic or signalling transcripts. Other SR proteins, hnRNPs, PTBP1, RBFOX2, ESRP1/2, QKI, SAM68 and DDX-family proteins can similarly function as context-specific oncogenes or tumour suppressors [48,49,68,69,70,71,72,73,74,75,76,77,78].

MYC provides a general mechanism for creating a splicing dependency. By increasing global transcription and the demand for RNA processing, MYC-driven cancers become sensitive to perturbation of core spliceosomal components. MYC also induces hnRNPA1, hnRNPA2 and PTBP1, shifting *PKM* splicing towards PKM2 and changing other growth-related transcripts [70,71,79]. RAS-MAPK and PI3K-AKT signalling alter transcription of splicing factors and phosphorylate SR proteins through CLK and SRPK kinases. These effects can occur within hours of treatment and may contribute to adaptive resistance before stable genetic resistance develops.

RBP localization is as important as abundance. Stress, phosphorylation, arginine methylation, and phase-separation behaviour influence whether an RBP is nuclear, chromatin-associated or sequestered in cytoplasmic granules. Mutations or post-translational changes that alter low-complexity domains can reconfigure the local concentration of splicing factors. Although this aspect is less mature than sequence-based models, it may explain why identical RBP expression levels can produce different splice outputs across cell states.

### 3.4. Epigenetic, Metabolic and Microenvironmental Remodelling

Hypoxia, nutrient stress, inflammation and therapy can remodel splicing without changing DNA sequence. Hypoxia alters RBP expression, DNA methylation and polymerase kinetics, and has been linked to changes in *VEGFA*, *MBD2* and other transcripts that promote angiogenesis or metastasis [72,73,74]. Non-coding RNAs can scaffold RBPs, compete for binding or alter their localization; examples include lncRNAs that regulate SRSF1-, SRSF6- or RAD51-dependent splicing and circular RNAs that engage RBFOX2 [75,76,77,78]. These interactions integrate splicing with broader RNA-regulatory networks.

Metabolism influences splicing through ATP availability, redox state, acetyl-CoA, S-adenosylmethionine and methylthioadenosine. Protein arginine methyltransferases modify Sm proteins and RBPs required for snRNP assembly and splice-site choice. Loss of *MTAP* causes methylthioadenosine accumulation and creates a selective dependence on PRMT5, linking a common metabolic deletion to spliceosomal vulnerability [80,81,82,83,84,85,86,87,88,89,90,91]. Conversely, splicing changes can remodel metabolism, creating a bidirectional feedback loop between RNA processing and nutrient adaptation.

The tumour microenvironment contributes additional signals. Cytokines, stromal contact, extracellular matrix stiffness, and immune pressure can change splicing-factor phosphorylation and transcription. Treatment itself is a strong selective event: kinase inhibitors, DNA-damaging agents, radiation and immunotherapy can induce transient isoforms that promote survival, while chronic exposure selects cells in which those isoforms become stabilized. Longitudinal sampling is therefore required to distinguish constitutive drivers from adaptive state markers.

## 4. Functional Consequences Across Cancer Hallmarks

### 4.1. Proliferation, Apoptosis and Growth-Factor Signalling

The most intuitive consequence of AS is the production of proteins with opposing functions. *BCL2L1* produces anti-apoptotic BCL-xL and pro-apoptotic BCL-xS, while *MCL1* generates a long survival-promoting isoform and shorter pro-apoptotic products [45,46]. Cancer cells frequently shift the balance towards survival through SRSF1 and related factors. Experimental splice switching of *BCL2L1* can restore apoptosis and enhance radiotherapy responses, demonstrating that isoform ratios can be functionally actionable [92,93]. *MDM4* poison exon inclusion provides another dosage-control circuit: defects in spliceosomal methylation can promote an NMD-sensitive *MDM4* transcript and activate p53 [44].

Growth-factor receptors are similarly affected. Exon skipping in *MST1R*/*RON* can generate a constitutively active receptor that promotes motility and invasion. *FGFR2* IIIb/IIIc switching changes ligand specificity and accompanies epithelial–mesenchymal transitions. *AXL* exon 10 skipping, regulated by PTBP1, promotes liver cancer invasion, while *FGFR3* variants can support aggressive prostate cancer phenotypes and docetaxel resistance [60,94]. *MET* exon 14 skipping is both oncogenic and clinically targetable [47]. These examples show why gene-level expression is often insufficient: a tumour can express the same total amount of a receptor while changing its signalling output through domain-selective splicing.

Splicing also controls downstream signalling proteins and tumour suppressors. SRSF1-dependent splicing of *MYO1B* and *PTPMT1* promotes gliomagenesis or breast cancer progression [68,69]. RAS itself is alternatively spliced into KRAS4A and KRAS4B, which differ in membrane-targeting sequences and metabolic dependencies; RBM39 degradation can alter KRAS4A and suppress cancer stem-like populations [95]. The functional importance of any single isoform nevertheless depends on relative abundance, protein stability, and pathway redundancy.

### 4.2. Metabolic Reprogramming and Stress Tolerance

The PKM1-PKM2 switch is the canonical link between splicing and cancer metabolism. MYC-induced hnRNP proteins and PTBP1 promote mutually exclusive selection of *PKM* exons, favouring PKM2 in many proliferating cells [79]. PKM2 supports flexible glycolytic and anabolic flux, although its precise role depends on enzyme activity, oligomeric state and lineage. SAM68, MTR4 and NEK2 can reinforce *PKM* splicing in lung adenocarcinoma, hepatocellular carcinoma and multiple myeloma [96,97,98]. These observations are best interpreted as part of a broader metabolic transcript program rather than a universal requirement for one isoform.

Alternative splicing affects mitochondrial proteins, lipid metabolism, amino-acid transport and redox homeostasis. Cancer cells under hypoxia or nutrient limitation can rapidly adjust domain composition and transcript stability without waiting for clonal selection. Intron retention is particularly relevant during stress because detained or retained introns can provide a reversible reservoir of incompletely processed RNA. Conversely, excessive intron retention can overload RNA surveillance and create proteotoxic or inflammatory stress, explaining why aggressive tumours may become dependent on high spliceosomal capacity [99].

Metabolic state also modifies the splicing apparatus. PRMT5-dependent methylation supports snRNP biogenesis and splicing fidelity; *MTAP* deletion lowers the effective margin of PRMT5 activity and creates susceptibility to MTA-cooperative inhibitors [84,85,86,87,88,89,90,91]. This is an example of collateral lethality in which loss of a metabolic enzyme exposes an RNA-processing dependency. Similar interactions may exist with one-carbon metabolism, NAD-dependent enzymes, and ATP-dependent helicases, but require careful separation of direct splice effects from general cytotoxicity.

### 4.3. Epithelial Plasticity, Invasion and Metastasis

Epithelial–mesenchymal transition (EMT) is accompanied by a coordinated splicing program. ESRP1 and ESRP2 maintain epithelial isoforms of *CD44*, *FGFR2*, *ENAH* and other adhesion or cytoskeletal genes; their suppression during EMT permits mesenchymal isoforms that favour migration and altered growth-factor responses [100]. RBFOX2, QKI, PTBP1 and hnRNP proteins contribute additional layers, and the resulting program is often reversible during metastatic colonization. This reversibility makes splicing a plausible mechanism for state transitions that occur faster than stable genomic evolution.

Individual events can have strong phenotypic effects. *AXL* exon skipping promotes invasion, while splice changes in *VEGFA* alter angiogenic signalling [74,94]. SRSF3-dependent *DDX5* processing and SRSF10-regulated *IL1RAP* isoforms support cervical cancer progression [101,102]. In pancreatic cancer, DHX38-dependent *RELL2* splicing restrains chemoresistance, whereas SF3A2-dependent *MKRN1* splicing promotes progression and cisplatin resistance in triple-negative breast cancer [103,104]. These studies illustrate that the same class of regulator can support or suppress malignancy depending on its target network.

Metastasis also creates an analytical problem: an isoform enriched in metastases may reflect a change in tumour-cell state, stromal composition or organ-specific normal tissue. Bulk RNA sequencing cannot reliably distinguish these possibilities. Single-cell and spatial isoform profiling, combined with lineage tracing and matched primary-metastatic samples, will be required to determine whether candidate events are cell-intrinsic and clonally maintained.

### 4.4. Genome Maintenance and Resistance to Therapy

Splicing regulates DNA repair, cell-cycle checkpoints and apoptosis, thereby shaping sensitivity to chemotherapy and radiation. Non-coding RNAs can modulate SRSF6- or SRSF1-dependent processing of *PICALM*, *RAD51* and *MCL1*, altering drug response [75,76,77]. Spliceosomal perturbation can produce R-loops, replication stress and DNA damage, creating opportunities for combination with PARP inhibitors or DNA-damaging agents. Pharmacological RBM39 degradation, for example, synergizes with PARP inhibition in high-grade serous ovarian carcinoma [105].

Therapy resistance may be driven by isoforms that delete a drug-binding or recognition domain. AR-V7 lacks the androgen receptor ligand-binding domain and predicts resistance to enzalutamide and abiraterone in metastatic prostate cancer [61,62]. *CD19* exon alterations can remove the epitope recognized by CAR-T cells, producing antigen escape after therapy [106,107]. Similar principles apply to receptor tyrosine kinases, apoptotic regulators and drug transporters. Because such isoforms may pre-exist at low abundance, treatment can select rather than create them.

Splicing can also mediate adaptive signalling. Kinase inhibition changes phosphorylation of SR proteins and can induce survival-promoting transcripts before genomic resistance emerges. Distinguishing causal resistance isoforms from passenger responses requires time-resolved perturbation, isoform-specific rescue, and testing in clinically relevant dose schedules. A strong biomarker should be measurable before therapy, change in the expected direction under treatment, and predict response independently of total gene expression.

### 4.5. Immune Regulation and Splicing-Derived Neoantigens

Alternative splicing shapes both tumour-intrinsic immune escape and immune-cell function. PD-L1, *SLAMF6*, *IL1RAP*, *IL1RN*, *DDX58* and *IRF1* can produce isoforms with altered membrane localization, signalling or cytokine responses [102,108,109,110,111,112]. In tumour cells, splice variants may change checkpoint expression, interferon sensing or antigen presentation. In lymphocytes and myeloid cells, the same regulatory machinery controls activation, exhaustion and differentiation. Therapeutic perturbation of splicing may therefore act simultaneously on cancer cells and the immune microenvironment.

A major conceptual advance is the recognition that aberrant splicing generates tumour-specific antigens. Retained introns, cryptic exons, alternative junctions and translation of nominally non-coding regions can create peptides absent from normal proteomes [40,41]. Computational predictions alone are insufficient because many abnormal RNAs undergo NMD or are not translated. Strong evidence requires tumour-restricted RNA expression, an open reading frame, proteasomal processing, HLA binding, immunopeptidomic detection, and T-cell recognition.

Recent studies have provided such evidence. Pan-cancer analyses identified recurrent neojunctions that are spatially conserved across tumour sites and naturally presented by HLA molecules; T-cell receptors recognizing *GNAS*- and *RPL22*-derived peptides can eliminate tumour cells [42]. In splicing-factor-mutant leukemias, recurrent SRSF2-induced mis-splicing of *CLK3* and *RHOT2* generates public neoantigens recognized by engineered T cells [43]. SNAF-based analyses further identified shared splicing neoantigens and tumour-specific transmembrane isoforms across cohorts [37]. These findings expand immunotherapy beyond DNA mutation burden and may be especially valuable in tumours with few coding mutations.

The opportunity is balanced by safety concerns. Low-level expression in an essential normal tissue, inflammation-induced expression, HLA restriction and antigen loss can all cause failure or toxicity. Normal-tissue atlases may miss rare cell types or developmental states, and junction abundance does not guarantee peptide presentation. Candidate public antigens therefore require deep normal-tissue RNA data, immunopeptidomics, functional T-cell testing and ideally multi-region confirmation of tumour-wide clonality.

### 4.6. Stemness, Lineage Plasticity and Tumour Ecosystems

Cancer stemness and lineage plasticity depend on rapid remodelling of interaction domains and signalling thresholds. Splicing offers a reversible mechanism for these transitions. KRAS4A, FGFR and CD44 isoforms can mark or support stem-like states, while poison exon networks tune the abundance of RBPs that maintain differentiation programs [95,100]. In MYC- or RAS-driven tumours, increased transcriptional load can create a dependency on spliceosomal induction, and recent work suggests that RAS-induced senescent tumour cells remain vulnerable to spliceosome targeting [70,113].

**Figure 3:**
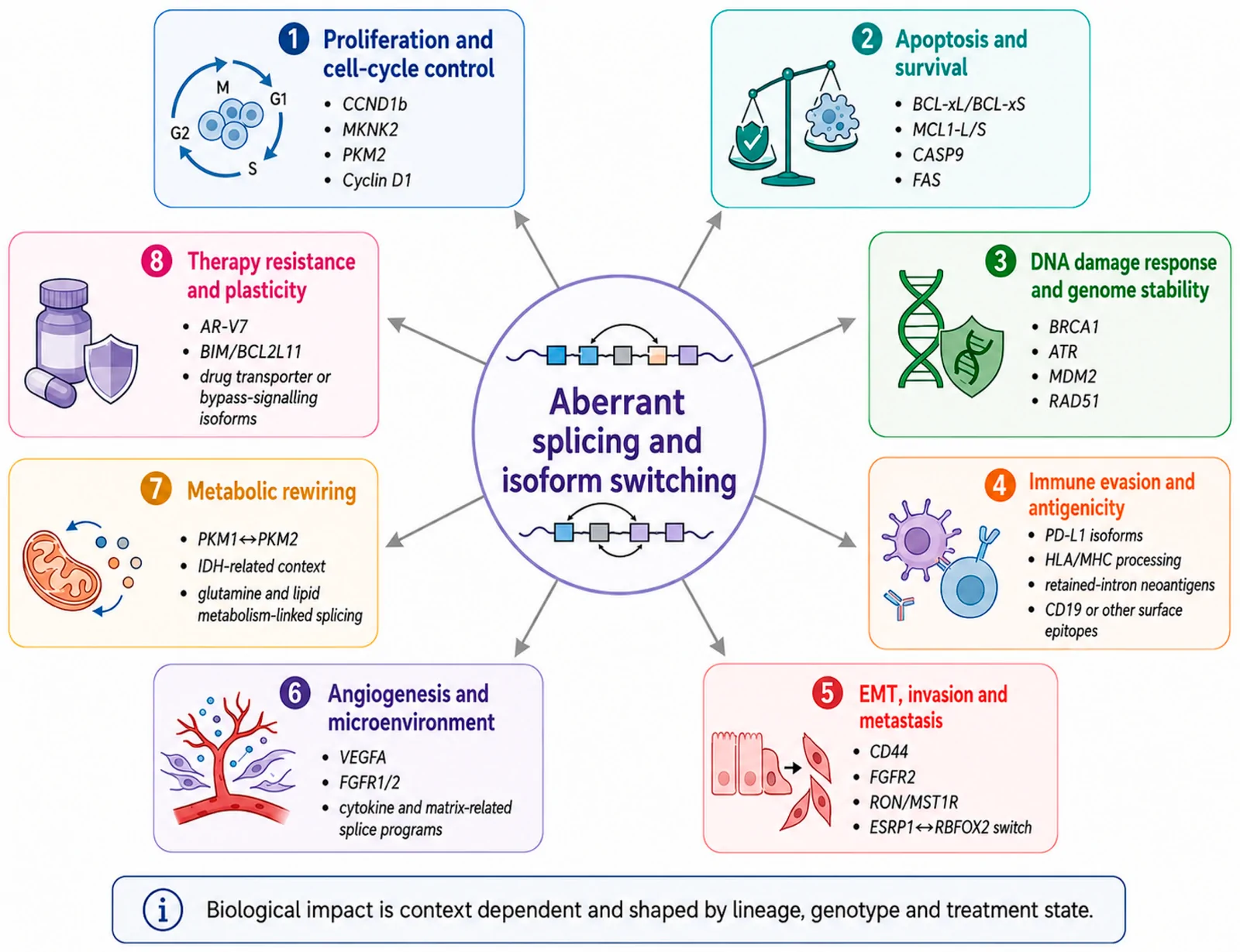
Functional consequences of aberrant RNA splicing across cancer hallmarks. Coordinated isoform switching remodels proliferation, apoptotic balance, genome maintenance, immune evasion, EMT and metastasis, angiogenesis, metabolic rewiring and therapy resistance. Representative genes are shown as examples, but the net effect of a splicing event depends on lineage, genotype, cell state, and treatment context. In the aberrant splicing schematic, the different colored boxes represent different exons.

At ecosystem level, stromal and immune cells have their own cell-type-specific splicing programs. Bulk tumour signatures can therefore reflect changing cellular composition rather than altered splicing within malignant cells. Single-cell long-read sequencing is beginning to separate these layers, revealing tumour-cell-state-specific isoforms and potential surface–intracellular target pairs [35,36,39]. The future unit of analysis is likely to be an isoform embedded in a particular cell state, genotype, and microenvironment, rather than an event averaged across an entire tumour. Collectively, the diverse functional consequences of aberrant RNA splicing across cancer hallmarks are summarized in Figure 3.

## 5. Representative Regulators and Cancer-Context Patterns

Cancer-associated splicing is organized by recurring principles rather than a single universal program. Spliceosome mutations are most prominent in hematological malignancies, whereas RBP dosage, oncogenic transcription and epigenetic state dominate many solid tumours. *SF3B1* mutations produce cryptic 3′ splice sites; *SRSF2* and *U2AF1* mutations alter sequence preference; *ZRSR2* loss impairs minor-intron splicing; and SRSF1, PTBP1 or hnRNP induction rewires networks downstream of MYC, RAS and lineage factors [19,20,21,22,23,24,25,26,27,65,66,67,68,69]. Table 1 summarizes representative examples.

The same regulator can have different phenotypic outputs across cancers because the available transcripts, co-factors and selective pressures differ. *SF3B1*-mutant cells in one lineage may lose a tumour-suppressive transcript through NMD, while another lineage translates an altered protein from a different cryptic site. Similarly, ESRP1 can suppress EMT in an epithelial tumour but support growth through specific receptor isoforms in another context. These observations argue against classifying splicing factors as intrinsically oncogenic or tumour suppressive without specifying the cellular system.

A practical framework is to distinguish driver events, enabling events and passenger events. Driver isoforms are sufficient and necessary for a malignant phenotype; enabling events create a permissive state such as increased stress tolerance or reduced differentiation; passengers report pathway activity but are not required. Functional classification should combine isoform-specific perturbation, rescue with splice-insensitive cDNA, protein-domain analysis, and in vivo validation.

## 6. Technologies for Detecting and Validating Cancer-Associated Splicing

### 6.1. Short-Read RNA Sequencing and Event-Level Analysis

Short-read RNA sequencing remains the most widely available approach for splicing analysis. Event-based tools such as rMATS, MAJIQ, LeafCutter, SUPPA2, and Whippet quantify exon inclusion or local splice variation using junction and exon reads [121,122,123,124,125]. Annotation-based methods are efficient for known events, whereas annotation-free junction clustering is valuable for cryptic sites and novel introns. The principal statistic is often percent spliced in (PSI), but PSI estimates become unstable at low read depth and may be confounded by changes in gene expression, transcript start sites, or polyadenylation.

Study design is critical. Adequate biological replication is more important than extreme sequencing depth for common events, while rare junction discovery requires both depth and stringent filtering. Batch, RNA integrity, library strandedness, read length, and tumour purity can all alter apparent splicing. Matched normal samples and a broad normal-tissue reference reduce false tumour specificity. For clinical FFPE material, targeted RNA panels or hybrid capture may provide more reliable junction detection than whole-transcriptome sequencing.

Differential splicing should be separated from differential transcript usage. A local exon change may occur within several full-length transcripts, and short reads cannot always determine which promoter, terminal exon, or coding sequence is connected to the event. Network and pathway analysis can identify coordinated programs, but causal interpretation requires isoform-level reconstruction or targeted experiments.

### 6.2. Long-Read Transcriptomics

Pacific Biosciences and Oxford Nanopore sequencing can capture full-length or near-full-length RNA molecules, directly linking distant exons, promoters, polyadenylation sites, and coding variants. This capability is particularly valuable in cancer, where complex transcripts, fusion isoforms and unannotated junction combinations are common [32,33,34,35,36]. Long reads can determine whether two local events occur in the same molecule, identify tumour-specific protein domains and improve design of isoform-specific assays or therapeutic oligonucleotides.

Long-read methods have distinct trade-offs. High-accuracy reads improve transcript identification, whereas high throughput improves abundance estimation. The LRGASP benchmark showed that read length and accuracy are more important than depth for transcript reconstruction, while deeper sequencing is advantageous for quantification; reference-guided tools generally perform best in well-annotated genomes, and rare novel transcripts require orthogonal evidence and replication [38]. Direct RNA sequencing preserves native RNA modifications and avoids reverse-transcription artefacts but currently has lower throughput and greater input requirements than cDNA approaches.

Novelty calls must be conservative. Truncated cDNA, template switching, internal priming and alignment errors can create false isoforms. Recommended practice includes replicate libraries, removal of incomplete molecules, transcript-level quality filters, comparison across multiple callers, short-read junction support and targeted RT-PCR or amplicon sequencing. A transcript observed once should not be treated as a therapeutic target merely because it is unannotated.

### 6.3. Single-Cell and Spatial Isoform Profiling

Conventional droplet single-cell RNA sequencing captures the 3′ or 5′ end of transcripts and is poorly suited to full-length isoform analysis. Smart-seq protocols improve gene-body coverage but have lower throughput and still depend on short-read reconstruction. Long-read sequencing of barcoded single-cell cDNA combines cell identity with transcript architecture, enabling analysis of malignant subclones, immune populations, and cell-state-specific splicing [33,34,35,36].

Recent computational frameworks recover cell barcodes and unique molecular identifiers from noisy long reads, collapse reads to consensus molecules and model splicing variation across cells or spatial spots [35]. Single-cell full-length profiling in colorectal cancer and glioblastoma has revealed thousands of previously unannotated isoforms, subtype-restricted transcript usage and candidate tumour-specific surface or neoantigen targets [36,39]. Importantly, isoform-level heterogeneity can exist even within a transcriptionally defined cell state.

Spatial isoform analysis is an emerging extension. It could identify junctions restricted to invasive fronts, hypoxic niches, or immune-excluded regions, but current spatial platforms have limited transcript coverage and resolution. A pragmatic strategy is to combine short-read spatial data for cell-state mapping with targeted or long-read validation in selected regions.

### 6.4. Proteogenomics, Immunopeptidomics and Orthogonal Validation

RNA abundance does not establish protein production. Proteogenomic workflows build customized protein databases from sample-specific junctions or full-length transcripts and search mass-spectrometry spectra for isoform-specific peptides. The strongest peptides span a novel junction or map uniquely to an alternative coding region. However, large customized databases increase false discovery, and many isoforms differ only in regions poorly detected by conventional proteomics. Parallel reaction monitoring, targeted antibodies and ribosome profiling can provide orthogonal evidence [8,40,41,42,43].

For neoantigens, immunopeptidomics directly measures peptides eluted from HLA molecules. Candidate selection should integrate RNA expression, reading frame, proteasomal cleavage, HLA binding and normal-tissue exclusion. Functional validation requires peptide-specific or tumour-reactive T cells, HLA-dependent recognition, and killing of cells that endogenously express the junction. The 2025 studies of public splicing neoantigens established a rigorous benchmark by connecting recurrent RNA events to natural presentation and T-cell cytotoxicity [42,43].

Routine molecular validation remains indispensable. Junction-specific RT-PCR confirms size and sequence; long-range PCR establishes full-length connectivity; minigene assays test cis-regulatory mutations; CLIP-seq maps RBP binding; and CRISPR or ASO perturbations test causality. Rescue experiments should use isoform-specific cDNAs that are resistant to the perturbation, and protein localization or activity should be measured when the event changes a functional domain.

### 6.5. Functional Screens and Causal Inference

Genome-scale CRISPR screens can identify spliceosome dependencies, but standard gene knockout does not reveal which isoform is responsible. Emerging strategies target splice sites, branch points, poison exons, or RBP motifs with CRISPR base editors, Cas13 systems, or pooled ASOs. These screens can be linked to single-cell RNA readouts to map perturbation-to-isoform-to-phenotype relationships. High-throughput minigene libraries and massively parallel splicing assays provide complementary measurements of cis-regulatory sequence.

**Figure 4:**
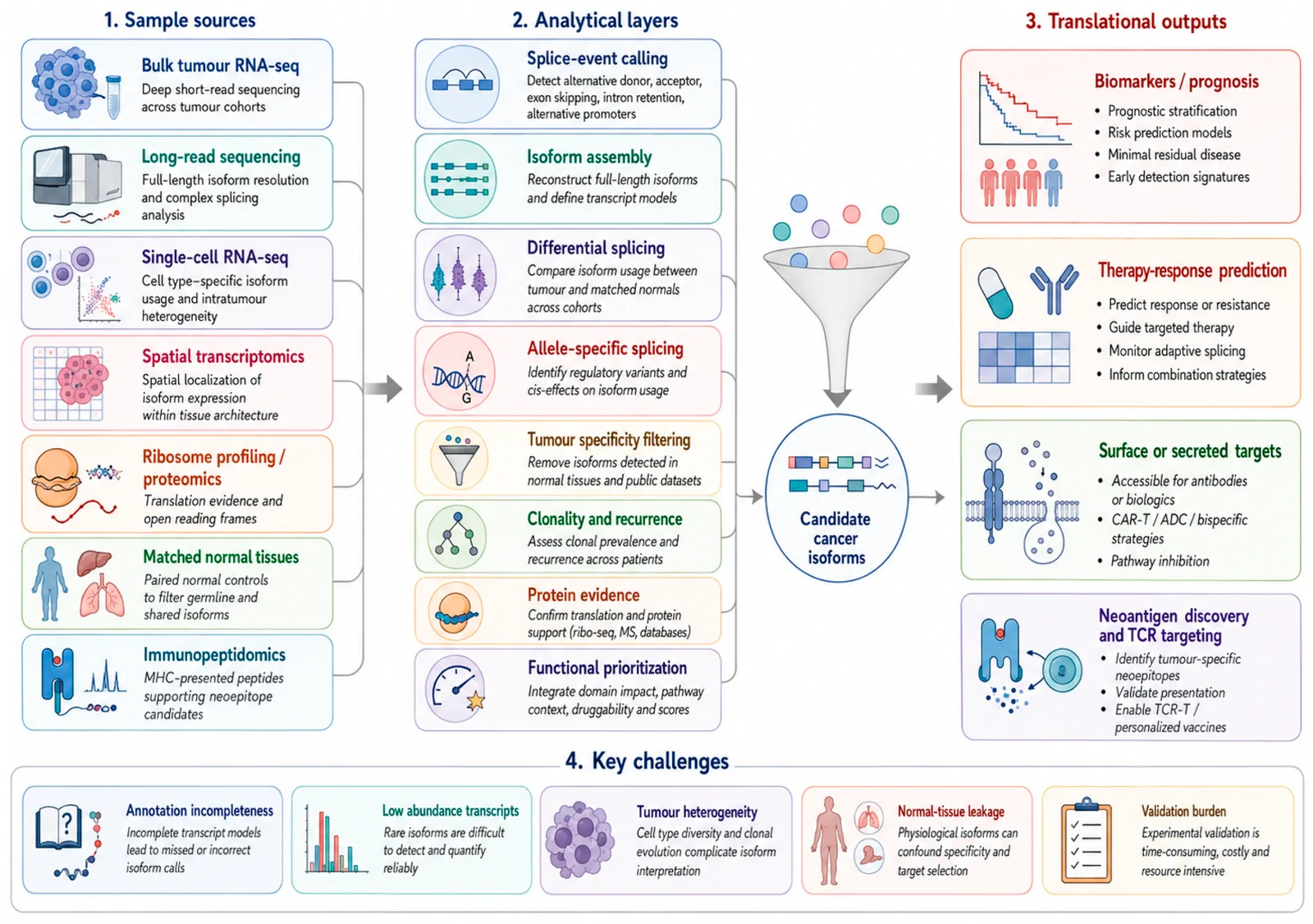
Technologies and translational workflow for isoform discovery, biomarker development and target nomination. Short-read, long-read, single-cell and spatial transcriptomic profiling, combined with proteogenomics, matched normal tissues and immunopeptidomics, define candidate cancer isoforms. These candidates are then filtered by tumour specificity, clonality, protein evidence and functional relevance to support biomarker development, response prediction, target nomination and neoantigen discovery, while challenges such as annotation incompleteness, low-abundance transcripts and tumour heterogeneity must be addressed.

Causal inference benefits from triangulation. A convincing cancer driver event should correlate with phenotype across patients, respond predictably to regulator perturbation, reproduce the phenotype when introduced, and be rescued by restoring the appropriate isoform. Where several isoforms change together, network-level models and combinatorial perturbation may be necessary. This is particularly important for core spliceosome inhibitors, whose antitumour effects rarely arise from one transcript alone. The principal experimental and computational approaches for isoform discovery and validation are summarized in Table 2, and their integration into a translational workflow is illustrated in Figure 4.

## 7. Splicing Biomarkers in Precision Oncology

Splicing biomarkers can be classified as diagnostic, prognostic, predictive or pharmacodynamic. Diagnostic markers include recurrent splice junctions that identify a tumour lineage or genomic lesion. Prognostic signatures combine multiple events associated with recurrence or survival, while predictive biomarkers identify patients likely to benefit from a therapy. Pharmacodynamic markers confirm target engagement by showing expected changes in sentinel transcripts after treatment.

Several clinically relevant examples already exist. *MET* exon 14 skipping is a predictive biomarker for MET inhibition, and AR-V7 in circulating tumour cells has been associated with resistance to androgen-receptor-directed therapy [47,62]. Splicing-factor mutations define biological subgroups of myeloid neoplasms and may enrich for sensitivity to spliceosome perturbation [19,20,21,22,23,24,25,26,27,63,64,126]. *MTAP* deletion is emerging as a biomarker for MTA-cooperative PRMT5 inhibitors, whose activity includes altered splicing and DNA damage [84,85,86,87,88,89,90,91].

The path from research signature to clinical assay is demanding. A biomarker must be analytically robust in the specimen type used for treatment decisions, whether fresh tissue, FFPE, blood or bone marrow. Junction-specific assays should define minimum read support, allele fraction, RNA quality and reporting thresholds. Isoform ratios may be more informative than absolute expression but are sensitive to degradation and cell composition. Prospective validation should show added value beyond genomic, histological and clinical variables.

Liquid biopsy is attractive because RNA junctions can provide high specificity, but circulating RNA is fragmented and low abundance. Exosomal RNA, platelet RNA and circulating tumour cells may enrich tumour-derived transcripts, whereas cell-free RNA offers broader sampling but greater background. Longitudinal measurements could detect adaptive isoforms before radiographic progression, provided the assay is sufficiently sensitive and standardized.

Composite biomarkers may be necessary for splicing therapies. A spliceosome mutation alone may not predict dependency; transcriptomic stress, RBP expression, NMD activity, *MTAP* status, MYC output and tumour proliferation may all contribute. Mechanism-based signatures should therefore integrate genotype with a functional splice readout rather than rely on a single mutation.

## 8. Therapeutic Targeting of RNA Splicing in Cancer

### 8.1. Modulators of the Sf3b Complex and Spliceosome Catalysis

Natural products including FR901464 derivatives, pladienolides, herboxidiene and meayamycin bind the SF3B complex and alter branch-point recognition [127,128,129,130,131]. Their potency established the spliceosome as a druggable target, but early clinical development of E7107 was limited by toxicity, including visual adverse events [132,133]. These experiences underscore the narrow therapeutic window of global spliceosome inhibition and the need for tumour-selective exposure, intermittent dosing or biomarker-defined sensitivity.

H3B-8800 was developed as an orally available modulator intended to exploit the increased vulnerability of spliceosome-mutant cells. Preclinical studies showed preferential lethality in mutant models, and a phase I study in myeloid neoplasms demonstrated target engagement but limited objective responses [63,64,126]. The results do not invalidate the strategy; rather, they suggest that genotype alone may be insufficient, drug exposure may need optimization, and appropriate clinical endpoints could include transfusion improvement or combination activity.

Core spliceosome perturbation can trigger R-loops, DNA damage, proteotoxic stress and innate immune signalling. In triple-negative breast cancer, spliceosome-targeted therapy induced an antiviral response and enhanced immune activity [134]. These pleiotropic effects create combination opportunities with DNA-damage therapies, checkpoint blockade and targeted inhibitors, but also increase the risk of toxicity in normal proliferative tissues.

### 8.2. Targeted Degradation of Rbm39

Aryl sulfonamides such as indisulam and E7820 act as molecular glues that recruit RBM39 to the DCAF15 ubiquitin ligase, causing proteasomal degradation and widespread splicing changes [114,115]. RBM39 participates in splice-site selection, transcription and protein interactions, and its loss preferentially affects transcripts with weak splice sites or specific sequence features [116,117]. The molecular-glue mechanism provides a route to pharmacologically remove an RBP rather than inhibit an enzymatic active site.

RBM39 degradation has shown activity in acute myeloid leukemia, neuroblastoma and ovarian cancer models, with potential biomarkers including DCAF15 expression, spliceosome genotype and lineage-specific dependencies [105,118,119,120]. E7820 produced RBM39 degradation in patients with splicing-factor-mutant myeloid malignancies, although clinical benefit was modest [120]. Rational combinations may be more effective: indisulam synergizes with PARP inhibitors in high-grade serous ovarian cancer and can alter KRAS4A splicing in cancer stem-like cells [95,105].

Resistance can arise through *DCAF15* loss, *RBM39* mutations that prevent recruitment, altered drug transport, or adaptive splicing networks. Monitoring degradation, sentinel splice events and resistance mutations in serial samples will be important. Molecular-glue discovery could in principle be extended to other RBPs, but successful compounds require a compatible ligase, a drug-induced protein interface and a tumour-selective dependency.

### 8.3. Protein Arginine Methyltransferase Inhibition

PRMT5 methylates Sm proteins and numerous RBPs, supporting snRNP assembly and RNA processing. Pharmacological inhibition disrupts splicing and can activate p53, DNA damage, and differentiation programs [44,80,81,82,83]. First-generation PRMT5 inhibitors showed antitumour activity but were constrained by hematological toxicity because PRMT5 is required in normal cells [87,88,89]. Type I PRMT inhibition can also perturb RNA-binding networks and may interact with PRMT5 pathways [80,81,90].

*MTAP* deletion creates a more selective therapeutic context. Loss of *MTAP* causes methylthioadenosine accumulation, which partially inhibits PRMT5 and sensitizes tumour cells to further reduction in PRMT5 activity [84]. MTA-cooperative inhibitors such as MRTX1719 and AMG 193 preferentially bind the PRMT5-MTA complex, increasing the therapeutic window in *MTAP*-deleted cancers. AMG 193 has produced preclinical regressions and early clinical responses in *MTAP*-deleted solid tumours, with aberrant splicing among its pharmacodynamic effects [91].

This strategy illustrates how splicing therapy can be biomarker-guided without requiring a spliceosome mutation. Key questions include whether particular splicing events mediate response, how resistance develops through *MTAP* restoration or pathway adaptation, and which combinations are tolerable. Preclinical data support combinations with chemotherapy, KRAS inhibitors, MAT2A inhibitors and DNA-damage therapies, but overlapping toxicity must be assessed prospectively.

### 8.4. Clk, Srpk and Other Signalling Nodes

CLK and SRPK kinases phosphorylate SR proteins and control their localization and activity. Inhibiting these kinases changes exon inclusion across a broad but distinct subset of transcripts. CLK2 and CLK3 have been implicated in breast cancer and cholangiocarcinoma, while small molecules such as SM08502 and related compounds show preclinical activity in gastrointestinal or MYC-dependent models [135,136,137,138,139]. Because kinase inhibitors are generally more chemically tractable than direct RBP inhibitors, this class offers opportunities for dose titration and combination.

Selectivity remains challenging. Different CLK and SRPK family members have overlapping substrates, and transcriptome-wide effects may vary by cell state. Pharmacodynamic assays should measure both SR-protein phosphorylation and sentinel splice changes. Other tractable nodes include RNA helicases, NMD factors, transcriptional kinases and RNA-modification enzymes, but each requires evidence that tumour dependency exceeds normal-tissue requirement.

### 8.5. Splice-Switching Oligonucleotides and Isoform-Specific RNA Therapy

Antisense oligonucleotides (ASOs) can bind splice sites or regulatory motifs and redirect exon inclusion without altering DNA. The clinical success of eteplirsen and nusinersen in inherited disease demonstrates that splice switching is feasible in humans [140,141]. In cancer models, ASOs have redirected *PKM* splicing, targeted *ERG* transcripts, and shifted *BCL2L1* toward pro-apoptotic isoforms [93,142,143]. Individualized ASO frameworks developed for rare genetic disease provide a conceptual route to patient-specific cancer junctions [144].

Cancer presents additional barriers. Efficient delivery to solid tumours is variable, repeated dosing may be required, and splice correction must reach a large fraction of malignant cells. Sequence specificity reduces systemic transcriptome toxicity but does not eliminate off-target hybridization, innate immune activation, or effects in normal tissues that express the target RNA. Chemical modification, conjugation, nanoparticles, and local delivery can improve exposure. RNase-H gapmers or siRNA can eliminate a pathogenic isoform when selective splice switching is not possible.

Decoy oligonucleotides can sequester a splicing factor away from its RNA targets, providing an intermediate strategy between global inhibition and transcript-specific ASOs [145]. Programmable Cas13 systems could degrade or edit selected RNAs, and small molecules that bind structured RNA may stabilize a desired splice conformation [146,147]. These approaches are promising but require highly resolved RNA structures and careful assessment of sequence-family off-targets.

### 8.6. Immunotherapies Directed Against Splicing-Derived Targets

Splicing-derived neoantigens create several therapeutic formats. Recurrent public peptides can support off-the-shelf vaccines or T-cell receptors, whereas private neojunctions may be incorporated into personalized vaccines. The demonstration of tumour-wide *GNAS* and *RPL22* neojunctions, as well as *SRSF2*-mutant leukemia antigens derived from *CLK3* and *RHOT2* mis-splicing, provides proof of principle [42,43]. SNAF analyses further suggest that shared splicing antigens and tumour-specific transmembrane isoforms may be common enough for systematic development [37].

Surface isoforms offer targets for antibodies, antibody–drug conjugates, bispecific molecules and CAR-T cells. A junction-specific extracellular epitope is particularly attractive because it does not require HLA presentation. However, full-length membrane topology must be confirmed, and low-level normal-tissue expression remains a major concern. Single-cell long-read studies can help prioritize isoforms that are both tumour-restricted and confined to malignant cells rather than stromal populations [39].

Combination with checkpoint blockade is rational because spliceosome perturbation can increase abnormal peptides and activate innate immune signalling [134,148]. Yet global splicing inhibition may also impair lymphocyte proliferation or antigen presentation. Dose, schedule and cell-type effects must therefore be optimized. A productive strategy may use transient tumour-directed splicing perturbation to increase antigenicity, followed by vaccination or adoptive T-cell therapy.

### 8.7. Rational Combinations and Resistance

The distributed nature of splicing suggests that combination therapy will often be required. Spliceosome inhibitors may enhance DNA damage and sensitize to PARP inhibitors or radiation; RBM39 degraders can interact with KRAS or DNA-repair pathways; PRMT5 inhibitors can combine with chemotherapy, KRAS inhibition, or MAT2A blockade; and ASOs can remove a specific resistance isoform while preserving the broader transcriptome [85,91,93,95,105,134]. Combinations should be selected from mechanistic evidence rather than simply screening for cytotoxic synergy.

**Figure 5:**
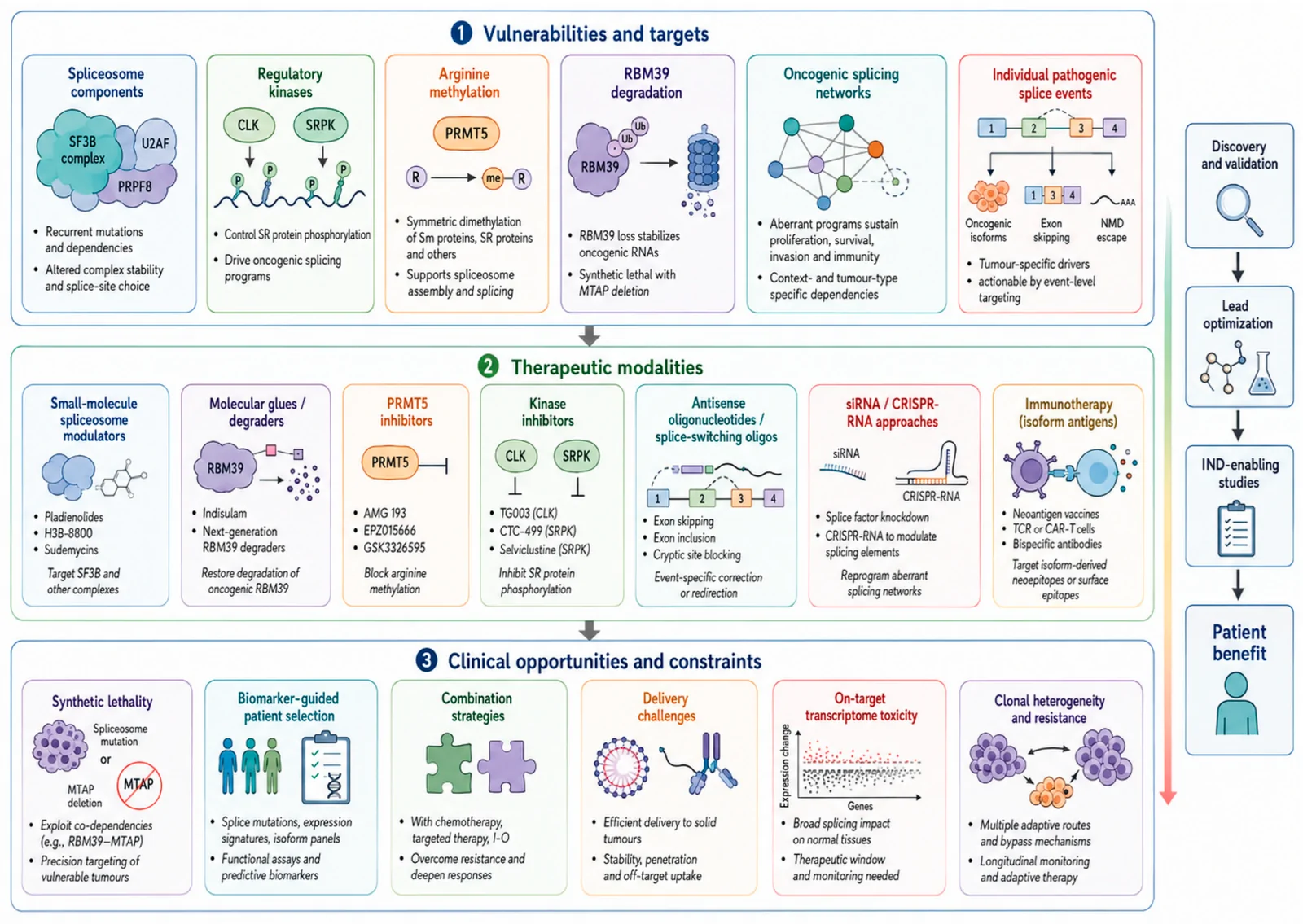
Therapeutic strategies targeting cancer-associated RNA splicing. Vulnerabilities span spliceosome components, regulatory kinases, arginine methylation, RBM39 dependency, oncogenic splicing networks, and individual pathogenic splice events. Therapeutic modalities include small-molecule splicing modulators, molecular glues or degraders, PRMT5 and kinase inhibitors, splice-switching oligonucleotides, siRNA or CRISPR-based RNA approaches, and isoform-directed immunotherapies; clinical translation requires biomarker-guided patient selection, combination strategies, and management of delivery, toxicity, heterogeneity, and resistance.

Resistance mechanisms include mutation or loss of the drug-recruiting ligase, restoration of methylation capacity, altered drug efflux, compensatory RBP expression, rewiring of splice-site choice, and clonal loss of the targeted isoform or HLA allele. Serial RNA sequencing is unusually informative for this drug class because it can measure both target engagement and adaptive transcriptome responses. The challenge is to distinguish pharmacodynamic changes from the events that actually drive resistance. An overview of these therapeutic vulnerabilities and intervention strategies is shown in Figure 5, while their mechanisms, development status, opportunities and limitations are summarized in Table 3.

## 9. Challenges and Future Directions

The first challenge is annotation. The human transcriptome is far from complete, but a permissive catalogue can become dominated by technical artefacts. Future studies should report transcript-confidence tiers based on replication, full-length support, junction quality, coding potential, and orthogonal validation. Community standards analogous to genomic variant classification would improve comparability across studies [32,35,38].

The second challenge is tumour specificity. Normal-tissue datasets differ in depth, cell composition and physiological state. Rare normal cell types, developmental tissues and inflamed tissues may express a candidate isoform that is absent from standard references. Single-cell normal atlases, direct testing in primary cells and highly sensitive targeted assays are needed before clinical development, particularly for T-cell therapies in which low-level expression can be dangerous.

The third challenge is heterogeneity. A bulk tumour event may be clonal, subclonal, state-specific, or stromal. Multi-region and longitudinal sampling should be integrated with single-cell or spatial analysis. Public neoantigens are especially valuable when tumour-wide, but clonality must be demonstrated rather than assumed from cohort frequency [39,42,43].

The fourth challenge is connecting RNA to protein and phenotype. NMD, nuclear retention, translational control, and protein degradation decouple transcript abundance from protein output. Proteogenomics and immunopeptidomics will become essential filters, but sensitivity remains limited. Functional evidence should prioritize isoform-specific perturbation and rescue, not only knockdown of the parent gene or regulator.

The fifth challenge is therapeutic index. Core splicing is essential, and even isoform-specific interventions may affect normal cells. Biomarker-guided selection, tumour-targeted delivery, intermittent schedules and synthetic-lethal contexts such as *MTAP* deletion can widen the window. Pharmacodynamic splice signatures should be incorporated early in trials to determine whether inadequate efficacy reflects lack of target engagement, insufficient dependency, or rapid adaptation.

A major future direction is multimodal isoform medicine. Short-read sequencing can provide sensitive junction quantification; long reads can define transcript architecture; single-cell and spatial assays can localize events; proteomics can confirm translation; and functional screens can establish dependency. Integrated models should prioritize not the most statistically significant event, but the event with the strongest combination of recurrence, tumour specificity, protein consequence, clonality and tractability.

Machine learning will contribute to splice prediction, but model performance depends on training data and biological context. Sequence-only models may predict the effect of a cis mutation, whereas trans-factor abundance, chromatin and cell state determine whether that prediction manifests in a tumour. Interpretable models that integrate sequence, RBP binding, polymerase kinetics and isoform-level single-cell data are likely to be more useful than purely black-box classifiers.

Several emerging regulatory dimensions further broaden this framework. A recent molecular cell study identified SMD2 as a pseudouridine reader that couples mRNA pseudouridylation to splice-site choice and tumorigenesis [149]. Transposable elements (TEs) are increasingly recognized as dynamic sources of regulatory DNA and RNA in gene-expression evolution and disease [150]. Recent cell studies further showed that TE exonization can reshape human interferon signalling through a decoy *IFNAR2* isoform [151] and can generate a broader reservoir of stable, translated, and functionally distinct protein isoforms [152]. Long-read analyses also demonstrate that transcription-start-site choice can constrain downstream splice-site and polyadenylation-site selection [153]. Together with established maps of recurrent cancer-associated splicing alterations [154], these observations support integrated models that connect RNA modification, TE biology, and transcription initiation with canonical spliceosome regulation.

Finally, clinical development should treat splicing as a dynamic phenotype. Baseline genomic lesions, treatment-induced splice changes, and acquired resistance should be measured together. Such longitudinal designs may reveal windows in which an adaptive isoform is transiently essential and therefore vulnerable to an ASO or combination therapy.

## 10. Conclusions

Alternative RNA splicing is a mechanistically diverse and clinically actionable layer of cancer biology. Genetic lesions, spliceosome mutations, RBP dysregulation and environmental signals converge on splice-site choice, generating isoforms that reshape signalling, metabolism, plasticity, immune recognition and treatment response. New long-read, single-cell and proteogenomic methods are converting local junction observations into full-length, cell-resolved and protein-validated models. At the same time, spliceosome modulators, molecular glues, PRMT inhibitors, kinase inhibitors, ASOs and splicing-neoantigen therapies are expanding the therapeutic repertoire. The decisive step for the field is not to catalogue more events, but to identify which isoforms are clonally conserved, functionally required, tumour-restricted and pharmacologically tractable.

## 11. Use of Generative Artificial Intelligence in Manuscript Preparation

During the preparation of this manuscript, the authors used ChatGPT (OpenAI, GPT-5.5 Thinking) mainly to assist with reference organization and schematic Figure design. The authors reviewed and edited all outputs and take full responsibility for the content of this publication.

## Figures and Tables

**Table 1:** Representative cancer-associated splicing regulators and isoform switches.

Category	Representative Alteration	Principal Splice Consequence	Cancer Context and Selected References
Core spliceosome	*SF3B1* hotspot mutation	Cryptic upstream 3′ splice sites; NMD or altered proteins	MDS, CLL, uveal melanoma and solid tumours [19,20,21,22,23,24,25]
Core spliceosome	*SRSF2* mutation	Altered motif preference and exon selection	Myeloid neoplasms; public mis-splicing neoantigens [19,25,43]
Core spliceosome	*U2AF1* mutation	Sequence-selective 3′ splice-site changes	MDS and AML [26,27]
Minor spliceosome	*ZRSR2* loss	U12-type intron retention	Myeloid malignancies [19,25]
SR protein	SRSF1 overexpression	*BCL2L1*, *MCL1* and oncogenic network switching	Breast, lung, pancreatic and brain tumours [65,66,67,68,69,92]
hnRNP/PTBP	MYC-hnRNP-PTBP axis	PKM2 and other proliferative isoforms	MYC-driven cancers [70,71,79]
Epithelial RBP	ESRP1/2 suppression	*CD44*, *FGFR2* and *ENAH* EMT program	Epithelial tumours and metastasis [100]
Receptor isoform	*MET* exon 14 skipping	Loss of CBL-binding region and receptor stabilization	NSCLC and other tumours; MET inhibitor sensitivity [47]
Hormone receptor	AR-V7	Loss of ligand-binding domain	Castration-resistant prostate cancer [61,62]
Immune escape	*CD19* exon alterations	Loss of CAR-recognized epitope	B-cell malignancies after CAR-T therapy [106,107]
Metabolism	PKM1-PKM2 switching	Glycolytic and anabolic adaptation	Multiple solid and hematological cancers [79,96,97,98]
Molecular glue target	RBM39 degradation	Broad sequence-biased splice disruption	AML, neuroblastoma, ovarian cancer [95,105,114,115,116,117,118,119,120]
Synthetic lethality	*MTAP* deletion-PRMT5 dependency	Reduced methylation reserve and splice stress	*MTAP*-deleted solid tumours [84,85,86,87,88,89,90,91]
Neoantigen	*GNAS*/*RPL22* or *CLK3*/*RHOT2* neojunctions	Naturally presented public peptides	Solid tumours and splice-factor-mutant leukemia [42,43]

**Table 2:** Experimental and computational layers for isoform discovery and validation.

Layer	Representative Approaches	Main Strength	Key Limitation
Short-read bulk RNA-seq	rMATS, MAJIQ, LeafCutter, SUPPA2, Whippet	Sensitive junction quantification in large cohorts	Limited full-length connectivity; depth and annotation dependence
Long-read RNA-seq	PacBio HiFi, ONT cDNA and direct RNA	Full-length isoforms, fusions and linked variants	Cost, input, truncation and novelty artefacts
Single-cell isoform profiling	Barcoded long-read cDNA, Smart-seq plus long reads	Cell-state and clone-specific isoform usage	Sparse molecules and barcode/UMI recovery
Spatial analysis	Spatial short reads plus targeted or long-read validation	Maps isoforms to anatomical niches	Limited transcript coverage and resolution
Proteogenomics	Customized protein databases, targeted MS, ribosome profiling	Evidence for translation and isoform-specific peptides	Low sensitivity and enlarged search space
Immunopeptidomics	HLA peptide elution and MS	Direct evidence of antigen presentation	HLA dependence and high input requirements
Mechanistic validation	Minigenes, CLIP, ASOs, CRISPR, rescue experiments	Tests cis/trans causality and phenotype	Throughput and model dependence
Clinical assay	Targeted RNA panels, RT-PCR, ddPCR, liquid biopsy	Standardizable and sensitive for selected events	Requires pre-specified targets and quality controls

**Table 3:** Therapeutic strategies targeting RNA splicing in cancer.

Strategy	Mechanism and Examples	Development Status	Main Opportunities and Limitations
SF3B modulation	H3B-8800; pladienolide and FR901464 derivatives	Preclinical to early clinical	Broad network effects and mutant-cell vulnerability; narrow therapeutic window [63,64,126,127,128,129,130,131,132,133,134]
RBM39 molecular glues	Indisulam and E7820 recruit RBM39 to DCAF15	Clinical proof of degradation	Potential lineage selectivity and combinations; DCAF15/RBM39 resistance [95,105,114,115,116,117,118,119,120]
PRMT inhibition	PRMT5, type I PRMT and MTA-cooperative inhibitors	Multiple early clinical programmes	*MTAP*-guided selectivity and splice stress; hematologic toxicity and adaptive resistance [80,81,82,83,84,85,86,87,88,89,90,91]
CLK/SRPK inhibition	Alters SR-protein phosphorylation and splice-site choice	Preclinical and early clinical	Chemically tractable; family redundancy and broad transcript effects [135,136,137,138,139]
Splice-switching ASOs	Mask splice sites or regulatory motifs	Approved in inherited disease; preclinical oncology	High sequence specificity; tumour delivery and repeated dosing [140,141,142,143,144,145]
RNA degradation/editing	Gapmers, siRNA, Cas13 and RNA-binding small molecules	Discovery to preclinical	Isoform-selective elimination; delivery and off-target challenges [146,147]
Splicing neoantigen therapy	Vaccines, engineered TCRs and adoptive T cells	Preclinical proof of concept	Public or personalized targets; HLA restriction and normal-tissue safety [37,40,41,42,43]
Surface isoform targeting	Antibodies, ADCs, bispecifics and CARs	Discovery to preclinical	HLA-independent; requires extracellular topology and stringent normal-tissue exclusion [37,39]

## Data Availability

No new data were created or analyzed in this study. Data sharing is not applicable to this article.
